# What is the impact of removing performance-based financial incentives on community health worker motivation? A qualitative study from an infant and young child feeding program in Bangladesh

**DOI:** 10.1186/s12913-021-06996-y

**Published:** 2021-09-17

**Authors:** Jeffrey Glenn, Corrina Moucheraud, Denise Diaz Payán, Allison Crook, James Stagg, Haribondhu Sarma, Tahmeed Ahmed, Adrienne Epstein, Sharmin Khan Luies, Mahfuzur Rahman, Margaret E. Kruk, Thomas J. Bossert

**Affiliations:** 1 Department of Public Health, College of Life Sciences, Brigham Young University, 2032 LSB, Provo, UT 84602 USA; 2 Department of Health Policy and Management, University of California Los Angeles, Fielding School of Public Health, Los Angeles, CA USA; 3 Department of Public Health, School of Social Sciences, Humanities and Arts, University of California Merced, Merced, CA USA; 4 Research School of Population Health, Australian National University, Acton, ACT 2601 Australia; 5 Nutrition and Clinical Services Division, icddr,b, Dhaka, Bangladesh; 6 Department of Epidemiology and Biostatistics, University of California, San Francisco, San Francisco, CA USA; 7 Department of Global Health and Population, Harvard T.H. Chan School of Public Health, Boston, MA USA

**Keywords:** Community health worker, Financial incentives, Motivation, Health workforce, Health systems, Child health

## Abstract

**Background:**

Community health worker (CHW) motivation is an important factor related to health service quality and CHW program sustainability in low- and middle-income countries. Financial and non-financial motivators may influence CHW behavior through two dimensions of motivation: desire to perform and effort expended. The aim of this study was to explore how the removal of performance-based financial incentives impacted CHW motivation after formal funding ceased for Alive and Thrive (A&T), an infant and young child feeding (IYCF) program in Bangladesh.

**Methods:**

This qualitative study included seven focus groups (*n* = 43 respondents) with paid supervisors of volunteer CHWs tasked with delivering interpersonal IYCF counseling services. Data were transcribed, translated into English, and then analyzed using both a priori themes and a grounded theory approach.

**Results:**

Results suggest the removal of financial incentives was perceived to have negatively impacted CHWs’ desire to perform in three primary ways: 1) a decreased desire to work without financial compensation, 2) changes in pre- and post-intervention motivation, and 3) household income challenges due to dependence on incentives. Removal of financial incentives was perceived to have negatively impacted CHWs’ level of effort expended in four primary ways: 1) a reduction in CHW visits, 2) a reduction in quality of care, 3) CHW attrition, and 4) substitution of other income-generating activities.

**Conclusions:**

This study provides new evidence regarding how removing performance-based financial incentives from a CHW program can negatively impact CHW motivation. The findings suggest that program decision makers should consider how to construct community health work programs such that CHWs may continue to receive performance-based compensation after the original funding ceases.

## Background

The World Health Organization Global Strategy on Human Resources for Health encourages the use of Community Health Workers (CHWs)—also known as “lay health workers” or “front-line health workers”—as key components of the health workforce, particularly in low- and middle-income countries facing shortages of professional clinicians [1]. A recent World Health Organization guideline defines CHWs as “health workers based in communities, who are either paid or volunteer, who are not professionals, and who have fewer than two years training but at least some training” [2]. While evidence demonstrates that CHWs can cost-effectively deliver high-quality services for a range of health conditions, establishing a successful CHW program requires complicated policy decisions regarding issues such as CHW training, management, and integration within health systems [1, 3–9]. The effectiveness of CHW programs relies heavily on the quality of the CHWs’ relationships with the communities they serve [10, 11]. Since CHW roles have expanded over the past few decades, an increasingly relevant challenge for program decision makers is how to optimize CHW performance without overburdening workers with tasks they lack the time and capacity to perform [12]. Another salient concern is maintaining high levels of CHW motivation [13].

**Fig. 1:**
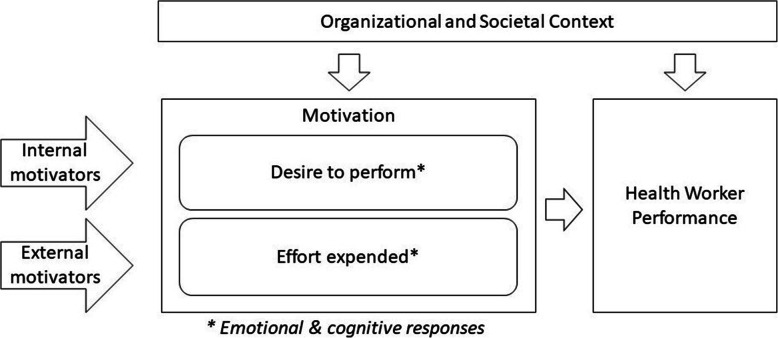
Conceptual Framework for Motivation and Health Worker Performance

Given the demonstrated association between CHW motivation and performance, questions surrounding CHW motivation have become central to health service quality and program sustainability as CHWs have become increasingly integrated into the global health workforce [11–15]. Figure 1 illustrates our conceptual framework for motivation and CHW performance. At the individual level, motivation can be conceptualized as a multidimensional construct shaped by both internal motivators—e.g., religious beliefs, moral standing, intrinsic values, etc.; and external motivators—e.g. financial incentives, material rewards, social recognition, etc. [13, 16, 17]. How these motivators affect workers depends on individuals’ emotional and cognitive responses (“motivation”), their organizational and societal contexts, and the interaction between these [18].

Various financial and non-financial compensation mechanisms exist to incentivize CHWs to complete their tasks and responsibilities. Financial rewards may change CHW behavior by influencing two dimensions of motivation: the extent to which workers adopt organizational goals (“desire to perform”) and the extent to which workers effectively work to achieve these goals (“effort expended”) [16, 19–21]. These two closely linked, yet distinct dimensions can exist independently or together. Intrinsic and extrinsic rewards can help maintain high levels of CHW motivation, via their “desire to perform,” by aligning CHWs’ competing interests with optimal job performance [10, 13, 17, 18, 22–27]. Although “effort expended” may also be influenced by rewards, this dimension of motivation is thought to be more heavily influenced by factors such as availability of resources and workers’ perceptions of their competency [18]. While the rich literature on motivation offers multiple ways to conceptualize motivation, “desire to perform” and “effort expended” provided a useful framework to approach this study’s research question.

While some CHW programs provide workers with regular salaries, others rely on a purely volunteer workforce or on various types and combinations of financial and non-financial factors, including intrinsic motivation, to maintain high CHW motivation and performance [13]. Evidence increasingly indicates that financial compensation improves CHW performance and retention [1, 10–13, 28–32]. Non-financial motivators and rewards are also considered important but are not recommended as a substitute for financial compensation [1]. Even in contexts where strong internal motivators are present, CHW motivation may still be buoyed by financial compensation that demonstrates the value of the work and reinforces existing motivation [24].

Compensation for CHWs can take the form of salaries (regular income) and/or performance-based incentives which tie payment to completing certain tasks or meeting certain benchmarks – and each of these may introduce unique challenges. Salaries were previously considered unsustainable in many low- and middle-income countries, although this assumption is recently being challenged [1, 23, 30]. Performance-based financial incentives may crowd out intrinsic motivation, encourage over-reporting of focus tasks, and lead to a substitution effect in which CHWs neglect unpaid tasks in favor of those for which they receive financial compensation [1, 10, 20, 25, 33, 34]. Moreover, performance-based financial incentives may not always be effective motivators if CHWs see them as insufficient, inconsistent, or unpredictable [13, 20, 35].

Although a number of studies have looked at the effects of financial compensation and non-financial motivators on CHW motivation, different circumstances will likely elicit varied responses [29, 32, 36]. Thus, there is still a need for additional empirical evidence in different contexts [10, 13, 23]. Studies on performance-based incentive programs in some settings have shown that withdrawing performance-based financial incentives can decrease health worker motivation, job attendance, and quality of care [37, 38], yet the evidence is particularly limited regarding the effects of removing performance-based financial incentives after introducing them to existing CHW programs in low-resource settings. This study addresses this gap by exploring how the removal of performance-based financial incentives was perceived to impact CHW motivation after formal funding ceased for an infant and young child feeding (IYCF) program in Bangladesh. A secondary research question looks at how inherent non-financial motivators (e.g., religious beliefs, values, social recognition, etc.) affected CHW motivation after financial incentives were removed.

## Methods

### Study context: Alive & Thrive Initiative in Bangladesh

The Alive & Thrive initiative (A&T) was a multi-component IYCF program implemented between 2009 and 2014 in Bangladesh, Ethiopia, and Vietnam, and funded by the Bill and Melinda Gates Foundation [39]. In Bangladesh, nationwide prevalence of stunting among children under 5 years was 36% in 2014 [40]. Child undernutrition in Bangladesh remains largely attributable to inadequate IYCF behaviors [41]. As one of four main A&T program components designed to improve IYCF practices in Bangladesh, interpersonal counseling services were delivered by the nongovernmental organization BRAC and its networks of nonpaid volunteer CHWs and paid health workers [42–44]. Volunteer CHWs included both Shasthya Shebikas (general community health volunteers) and Pushti Shebikas (community health volunteers working exclusively in IYCF) who worked in their home communities, and paid health workers included Shasthya Kormis (general paid health workers) and Pushti Kormis (paid health workers working exclusively in IYCF) who covered a larger area than the volunteer workers [35, 45–47]. Prior to A&T, the general community health volunteers delivered health education messages about breastfeeding as part of the standard service package within BRAC’s Essential Health Care Program [45]. Upon initiation of A&T, the IYCF-focused community health volunteers and IYCF-focused paid health workers were introduced in areas not covered by existing general community health volunteers and general paid health workers, to strengthen IYCF services. During A&T, the volunteer CHWs began receiving performance-based financial incentives (6 to 8 USD per month compared to a mean monthly household expenditure of about 106 USD) and additional training to deliver enhanced messages, personalized counseling, and hands-on support related to early and exclusive breastfeeding and appropriate complementary feeding practices [45, 48, 49].

A cluster-randomized program evaluation demonstrated that A&T’s combined approach in Bangladesh increased exclusive breastfeeding by 36.2 percentage points, early initiation of breastfeeding by 16.7 percentage points, and minimum dietary diversity by 16.3 percentage points [43, 50]. When A&T ended in December 2014, project support ceased for IYCF-specific financial incentives, refresher trainings, or job aids, although BRAC still expected IYCF counseling and education services to continue. While some A&T program activities and benefits continued, other activities, such as training and reporting, ceased altogether or experienced a substantial attenuation in implementation [51]. For example, 2 years after A&T ended, exposure to volunteer CHWs for interpersonal counseling was higher in intensive intervention areas than comparison areas; however, it had still experienced a decrease from 87.4% to 59.3% in intervention areas [44]. While this decline in program activities is likely a result of multiple factors and complex processes, the objective of the present study is to focus specifically on how the loss of financial incentives contributed to this decline via CHW motivation.

### Study sample and data collection

As part of a larger effort to assess the sustainability of A&T in Bangladesh, focus groups were conducted with representatives from multiple sub-districts in which A&T was implemented [51]. Seven districts were purposively selected to achieve geographic variability from a sample of 10 districts that had participated in a previous A&T endline evaluation. Each focus group consisted of male and female BRAC employees from multiple sub-districts within the selected district who were purposively selected because of their experience working as paid supervisors of CHWs tasked with delivering IYCF services. The focus groups were held with supervisors because an objective was to collect data on managerial-level insights on program implementation, and supervisors were likely to provide a program-level perspective that is broader than the views of individual CHWs. The research team initially planned for seven focus groups but created a contingency plan for additional focus groups if data saturation was not reached. All participants were a minimum of 18 years of age and provided verbal consent to participate in the study.

A semi-structured focus group discussion guide was developed by the research team and used to elicit perspectives from BRAC employees about the perceived impact of removing A&T financial incentives from volunteer CHWs as well as on other post-implementation related programmatic issues (e.g., implementation challenges, continuation of activities, adaptations, funding, human resources, and quality of services). The guide was developed in English, and after an iterative revision process, translated into Bengali.

Focus group discussions occurred between January–May 2017, 2 years after A&T program support ceased, and were conducted in Bengali by experienced male and female facilitators who received specific training about this study. The focus group facilitators had academic training in qualitative research and the social sciences and were employed as full time researchers at the time of the study. After receiving approval from BRAC to organize the focus groups, the research team contacted the participants via email to explain the study and invite them to participate in the focus group. Participants received informed consent forms with information about the study purposes, methods, and privacy. All focus groups were audio recorded with permission from participants. A designated note taker made field notes during the discussions. Focus groups occurred in training rooms at the local BRAC offices and lasted between 90 and 160 min. This study was reviewed and approved by Institutional Review Boards at the Harvard T.H. Chan School of Public Health, University of California Los Angeles, and icddr,b in Bangladesh.

### Data analysis

Audio recordings were transcribed verbatim, and the transcripts were translated into English before being uploaded to NVivo (QSR International Pty Ltd. Version 12, 2018). The codebook was developed using a combination of inductive and deductive techniques. Two parallel components of health worker motivation—“desire to perform” and “effort expended”—were themes identified a priori, which helped to identify sub-themes on the impacts of removing financial incentives and CHW motivation [16, 19, 20]. The research team used a deductive approach to identify emergent themes [52] based on participants’ responses that explicitly mention how they viewed the relationship between incentives and CHW motivation, including types of non-financial motivators identified in prior CHW studies [10, 13]. Emergent codes were discussed and defined to generate a final codebook. Two researchers used the final codebook to independently code all of the transcripts. Coding discrepancies and preliminary findings were iteratively discussed by the research team to achieve consensus.

## Results

Seven focus groups were conducted across seven districts (*n* = 43 respondents; group size range: 6–7 participants). Participants (see Table 1) included both men and women between the ages of 24 and 36 years. All participants had a master’s degree and between 2 and 6 years of work experience in their roles as CHW supervisors, meaning that they all had experience working on A&T implementation before the project ended.

**Table 1:** Demographic Characteristics of Focus Group Participants

Total N of Participants	43
N Male	32
N Female	11
Mean Age in Years (range)	33.1 (24–37)
Mean Years of Experience (range)	4.7 (2–6)
Master’s Degree	43

According to most respondents, removal of performance-based financial incentives was perceived to have negatively affected volunteer CHW motivation in terms of “desire to perform” and “effort expended” on IYCF-related tasks. Supervisors in all of the focus groups reported observing a direct negative impact on volunteer CHW motivation as a result of removing financial incentives to IYCF tasks. A small number said they had yet to see these effects in their own sub-districts. A respondent in one focus group reported that in one sub-district, volunteer CHWs were still receiving small performance-based financial incentives from other sources for their IYCF activities, but they had been notified that those incentives would be discontinued at the end of the year. One respondent from a different focus group shared that volunteer CHWs had the expectation that incentives would be paid to them in the near future. Even in these cases, supervisors expected a decrease in motivation once financial incentives completely ceased. Specific types of negative effects and illustrative quotes are included in Table 2. These effects are discussed in more detail below in addition to non-financial motivators. All quotes are from sub-districts in which the incentives had completely ceased.

**Table 2:** Key Themes related to the Effects of Removing Performance-Based Financial Incentives on Community Health Workers’ (CHWs) Motivation

CHWs’ Motivation	Perceived Effect	Illustrative Quote
Desire to Perform	Decrease in Desire to Work without Financial Compensation	“Incentives must be there; otherwise you cannot get the job done.”
	Change in Pre- vs Post-Intervention Motivation	“Stopping the incentive after giving them regularly, it is the problem. If the [financial incentive] provision was not there, there might be no trouble.”
	Household Income Challenges Due to Dependence on Incentives	“They (the volunteer CHWs) consider this money as salary. They are poor people. ‘Earlier we got 500, 700, 1000 taka. Now [we are] getting 200, 150 taka.’ They told us to take care of this issue, at least if it could stay the same.”
Level of Effort Expended	Reduction in CHW Visits	“Now, the Shebikas (volunteer CHWs) are reducing the visit count due to the reduction on the incentives from the last 2 months.”
	Perceived Reduction in Quality of Care	“But now, no incentives are given to them. They are not delivering the message properly.”
	CHW Attrition	“Now they deliver the messages but do not get any incentives, this is [why] the Shebikas (volunteer CHWs) are dropped out.”
	Substitution of Other Income-Generating Activities	“Shebikas (volunteer CHWs) cannot get anything from here (IYCF counseling). They now focus more on nutritious issues (i.e., selling micronutrient powder … There’s a little more work here.”

### Effects of removing performance-based financial incentives on CHWs’ motivation: decreased desire to perform

Respondents reported three primary effects from removing performance-based financial incentives on volunteer CHWs’ desire to perform, namely: 1) a decreased desire to work without financial compensation, 2) changes in pre- and post-intervention motivation, and 3) household income challenges due to dependence on incentives.

The first theme, a decrease in volunteer CHWs’ individual desire to conduct IYCF activities, was discussed in all of the focus groups. While supervisors reported that many CHWs were still conducting IYCF visits, they believed IYCF activities had become a lower priority after the removal of financial incentives, as stated in the following quote: *“Health workers are showing their reluctance for the reduction of money.”*

Supervisors explained that volunteers CHWs generally no longer wanted to deliver services without financial compensation. In one supervisor’s words, *“The mentality [of the] service is coming to people economically. ‘I will not work here without money.’ One will give you one or two days free service. Next time, if you want the service, that person will expect some benefit.”*

A second noted effect was a change in volunteers CHWs’ motivation from before A&T introduction to after A&T ceased. As mentioned with the first theme, volunteer CHWs experienced a decreased desire to perform once financial incentives ceased, but beyond that, some supervisors believed introducing and then removing the financial incentives caused volunteer CHWs to have less motivation than they had before the A&T program began. This resulted from an apparent change in volunteer CHW expectations regarding how they felt they should be compensated for IYCF tasks once they began receiving financial incentives—*“Whatever we say, budget is involved with it. Earlier, they got honorarium. If I get honorarium once, I will not go there again without honorarium.”*

A third effect on volunteer CHWs’ desire to perform consisted of household income dependence on financial incentives during A&T implementation and the resulting income challenges that arose once financial incentives were removed. Over the 5 years of A&T implementation, volunteer CHWs came to see the financial incentives as a vital source of income on which their families depended and to meet their basic needs. Supervisors noted this dependence and the ensuing dissatisfaction among volunteer CHWs once financial incentives were removed.

Supervisors in one focus group discussed how the increased opportunity cost of completing IYCF tasks without financial incentives prevented volunteer CHWs from performing their IYCF counseling responsibilities. One participant explained that if a volunteer CHW is compensated for making an IYCF visit it might be worthwhile for the husband to stay home and take care of the animals and house. However, without a financial incentive for the wife, she would need to stay home so the husband could earn sufficient income for the family to meet their basic needs:

*She will … tell her husband that [he does] not need to go to work today. ‘Stay home today. [You] do not need to go to work. You can earn 300 taka. I will get 2000 taka.’ And then she will tell her husband to guard the house. … If [there was] an incentive, then she [would] willingly come here.*

### Effects of removing performance-based financial incentives on CHWs’ motivation: level of effort expended

While, as described above, changes in the availability of financial incentives affected volunteer CHWs’ level of desire to perform IYCF activities, the focus group data also provides evidence that removing financial incentives directly influenced volunteer CHWs’ level of effort. Respondents reported four negative effects on volunteer CHWs’ level of effort expended, namely: 1) a reduction in volunteer CHW visits, 2) a perceived reduction in quality of care, 3) volunteer CHW attrition, and 4) substitution of other income-generating success of the A&T model relied on volunteer CHWs regularly visiting their communities to provide education and follow-up about IYCF to mothers and pregnant women. Supervisors reported that, after incentives were removed, volunteers CHWs began making less frequent IYCF visits and, in some cases, even stopped conducting IYCF visits at all—*“The Shebika (volunteer CHW) who was living around is visiting once [per month]. When there was incentives … the Shebika (volunteer CHW) visited several times [per month] because if they visit then they could get the incentive. This is the impact.”*

Due to the volunteer nature of the program, supervisors felt limited in terms of what they could do to ensure the visits. As one supervisor noted, *“If the Shebika (volunteer CHW) does not visit, we cannot ask them why they are not going. They are not our salaried staff. If they do not visit we cannot force them. And the Shebika (volunteer CHW) will not visit without any incentives.”*

Even when visits continued to be conducted, there was widespread agreement among the supervisors that the quality of services had decreased due to the removal of financial incentives. Supervisors made several similar comments regarding this issue, including *“Health workers … have been a little sloppy”* and *“The Shebika (volunteer CHW) thinks that ‘If the job was a paid one, then I could have done better.’”*

Going beyond mere “sloppiness” or not doing the job as well as possible, some supervisors pointed out specific ways they believe the decrease in service quality was impacting IYCF beneficiaries:

*They (volunteer CHWs) find out who are the newborn babies because if they can ensure colostrum feeding then she could earn 50 taka as an incentive. … Before she (volunteer CHW) prepared the note [indicating] in which household there is a newborn baby or which mother is ensuring the breastfeed within one hour of birth. … It is known [now] that she is not working properly because she does not get incentive now.*

Another supervisor commented, *“Maybe a few (volunteer CHWs) are doing, maybe taking the notes about the newborn babies, but due to that less attention, our coverage has already been decreased.”*

Supervisors in all but one focus group noted a transfer of responsibility to the paid health workers, who were now being required to take on the work that volunteer CHWs were no longer willing to do—*“Since the incentive is stopped or somewhat incentive is less so their (volunteer CHWs’) tendency for working in the field is getting down. The Pushti Kormis (IYCF-focused paid health workers) are doing the tasks of Shebikas (volunteer CHWs).”*

The result was a decrease in service quality since the paid health workers were under considerable amounts of pressure and were unable to effectively manage the increased load of both jobs—*“Now the Pushti Kormi (IYCF-focused paid health worker) has to find out the pregnant women, the newborn babies of one area, but if the Shebika (volunteer CHW) is paid incentives then she could complete those tasks [to] find out the pregnant mothers and could inform about the newborn babies.”*

Additionally since the paid health workers were not village locals, it was much less convenient for them to regularly visit the mothers for counseling and follow-up. One supervisor explained, *“Since the Shebika (volunteer CHW) resides in the same area, they can monitor the overall situation, But the Pushti Kormi (IYCF-focused paid health workers) visit once a month.”* Another quote further described this effect:

*When they drop out then our workforce is reduced, there becomes a gap in between the message delivery, the Pushti Kormi (IYCF-focused paid health workers) cannot deliver the messages while visiting 4-5 days. The Shebika (volunteer CHW) belongs to that village and she will work in that village only, so she can visit every day and there will be no gap in between the times.*

The supervisors believed this negatively impacted IYCF service quality:

*The health worker (IYCF-focused paid health worker) goes once a month, where the Shebika (volunteer CHW) was staying around the house and because of the incentive, she could not go anymore, which does not go away. There is a difference between telling a mother once and saying repeatedly.*

In addition to volunteer CHWs completing fewer visits and delivering lower quality services, supervisors in the majority of focus groups explained that volunteer CHWs were quitting their positions because financial incentives were no longer available. Volunteer CHW attrition was seen to impact IYCF services on a broad scale because of the significant drop in numbers of trained volunteer CHWs*—“We stopped giving the incentive in 2016. Now there are 60-65 Shebikas (volunteer CHWs) out of 280 [previously].”*

In comparison, attrition had not been identified as a problem during A&T implementation when incentives were available, as one supervisor observed, *“Since the incentive was more at that time there were no drop out of Shebikas (volunteer CHWs).”*

A final noted effect from removing volunteer CHWs’ financial incentives for IYCF services, and a key reason for the decrease in time dedicated to IYCF services, was that many volunteer CHWs were now dedicating more time to other tasks for which financial incentives are still being offered. For example, supervisors in most focus groups discussed how volunteer CHWs in their sub-districts had shifted from delivering IYCF services to selling nutrition packets (Pushtikona) in order to generate additional income.*—“[The] main target is to sell the Pushtikona, so they give less time in demonstrations or other activities. Very little time was given to children aged 0-6 months.”* Another supervisor asserted, *“They are automatically saying that they will sell more Pushtikona, since they are being paid for that, but they will not give the demo [about complementary feeding], do not find the pregnant women. If we ask them why, then they say that we were not paid in the last month.”*

### Non-financial motivators and CHW motivation

Some of the focus groups discussed the effects of non-financial motivators on CHW motivation. In this context, these non-financial motivators were not provided deliberately by A&T as performance-based rewards; rather, they were internal and external factors that existed due to the nature of the CHW work within the target communities. However, supervisors described how they used these factors as a strategy to motivate volunteer CHWs to dedicate more effort towards their jobs. In contrast with the financial incentives, these motivators were not discontinued after A&T funding ceased.

When asked about effective strategies to motivate volunteer CHWs, supervisors described how reminding them of the important nature of the task and their community ties could have an impact:

*We say “If the mother does not do the exclusive breast feeding till 6 months then it will harm the baby who might be your nephew or niece, may be your cousin’s brother or cousin’s sister. If you do this small help to them, the mother will pray for you. At least continue this sacred task, after that whenever we will get the money we will provide it from office.”*

Another supervisor remarked, *“Convince and motivate them by saying that this is a work for humanity by which your acceptance to the people will increase.”* As noted in the previous comment, social recognition was another non-financial motivator that supervisors believed was helping to maintain motivation and drive performance among some volunteer CHWs who continued to work diligently after the removal of the financial incentives. A supervisor explained, *“Motivation is above all. We tell them that others cannot do what you have done. ‘Although I am more educated than you, I cannot take your position in your area. … All know you by your name.’ I motivate them in all ways.”*

Supervisors from multiple groups commented on the importance of recognition within the community: *“You are known to the villagers due to the effective service you are providing to them.”* and *“Shutting down of the incentives is [having] little effect. [*Volunteer *CHWs] are moving forward on the basis of the popularity that has been working before.”*

Supervisors also believed that encouraging volunteer CHWs to feel a sense of responsibility motivated them to continue working without a financial incentive*—“She was given [the] responsibility. That way she is doing the job.”*

Despite these efforts, some supervisors described how intrinsic motivation alone is insufficient without financial compensation:

*How long we should convince them (volunteer CHWs)? If someone tells me that, “You work for BRAC and you will get the chance to achieve the heaven very easily,” I will never work for them. The head office, our superior officers, also know that they have no salary except the incentives, so the Shebikas (volunteer CHWs) should be brought by engaging them by using any strategy with income generating tasks because there is a strong relation between the work and money.*

## Discussion

Removing performance-based financial incentives from a volunteer CHW program in Bangladesh was perceived to have negative effects on CHW motivation. Similar to findings from previous research, this study demonstrates that CHWs may experience a decreased desire to fulfill their jobs following removal of performance-based financial incentives, and that a major contributing factor was the crowding out of intrinsic motivation [26, 38]. The IYCF program saw decreases in quality of care and number of visits as CHWs dedicated less effort to their jobs and increasingly substituted income-generating activities for IYCF responsibilities after financial incentives were removed [25, 33]. Previous studies conducted among comparable populations of CHWs in Bangladesh similarly found that financial compensation was a strong source of motivation and the main factor associated with CHW retention [31, 53]. The voluntary nature of CHW programs, in addition to other contextual factors, is important, as studies in which performance-based financial incentives were introduced to salaried health workers in different contexts found that incentives had little to no effect on worker motivation [54, 55].

Previous studies on the A&T program in Bangladesh found that performance-based financial incentives were an important element to the program’s success in achieving its IYCF aims. In one analysis, these incentives were directly associated with improved quantity and quality of IYCF service delivery [56]. Other impact evaluations of the A&T program found that an intensive IYCF intervention package that included performance-based financial incentives had significant positive impacts on breastfeeding practices, maternal nutrition among pregnant women, and child development compared to a nonintensive intervention package that did not include financial incentives [43, 57, 58]. Our finding that CHWs experienced a decrease in motivation post-incentive removal bolsters the conclusion of a separate study on the A&T program in Bangladesh that the removal of cash incentives was likely a contributing factor to reduced program exposure and impact after program funding ceased [44]. However, it is critical to note that the negative effect of removing financial incentives on CHW motivation may be only one of many contributing factors that affected IYCF service delivery in A&T intervention areas after the program ceased. Attributing a specific effect size to financial incentive removal in comparison with other factors is beyond the scope of this qualitative analysis.

Although the main findings are consistent with existing studies, our qualitative data highlights perceived effects of removing incentives that are less prominent in the CHW literature. First, some supervisors believed that CHW motivation was lower following removal of the performance-based financial incentives than it was prior to the introduction of incentives at program onset. While this perspective was mentioned only occasionally and was not explored in depth during the focus groups, if true, this finding has important sustainability implications for global health programs considering introducing performance-based financial incentives where they do not currently exist. This new financial compensation structure could potentially undermine existing non-financial motivational mechanisms for CHWs, including a commitment to serve their local communities.

Programs must consider how volunteer CHWs may be uniquely affected by performance-based incentives as these represent the sole source of financial compensation for their work. Given that the volunteer CHWs had grown dependent on IYCF financial incentives as a vital source of income, removing the incentives could have affected various aspects of their lives and well-being—creating a sense of dissatisfaction with the available incentives with which an accompanying decrease in motivation would be expected [11, 12]. Not only did CHWs lose the ability to purchase food or pay for school supplies, but they were also forced to adjust familial roles—i.e., breadwinner vs homemaker—to make up for female CHWs’ lost income. In such cases, the reduction in CHW visits may be due less to a lack of desire to perform and possibly due to the practical realities of meeting a family’s basic needs. This finding reinforces the idea that financial remuneration is even more critical in high-poverty areas in order to reach a point at which intrinsic motivation can also play a role [20, 26]. Of course, the magnitude of the financial incentive relative to household expenditure is an important factor in determining the effects of withdrawing the incentive. In Bangladesh, the mean monthly household expenditure in the A&T intervention areas was around 9000 taka (106 USD), and the monthly incentive for CHWs typically totaled between 500 and 700 taka (6 to 8 USD) [48, 49]. We expect that withdrawing a financial incentive that represents a larger percentage of household expenditure than this would be more demotivating for CHWs and more disruptive to familial roles.

Finally, in some sub-districts CHWs continued IYCF activities despite no longer receiving financial compensation. This suggests not a ‘crowding out’ but a ‘crowding in’ of intrinsic motivation, which may be more likely to occur in programs where CHWs feel more support—e.g., training, encouragement—from their supervisors and more confident in their own abilities [24]. Although this study was unable to assess levels of support and confidence among those CHWs who continued delivering services after A&T financial incentives were removed, supervisors believed that it was unlikely that CHW motivation would remain high without reinstituting financial incentives in some form.

While this qualitative study adds significant understanding to the effects of removing performance-based financial incentives on CHW motivation, there is a need for larger scale, well-designed studies that use mixed-methods research to address the same question. The findings from this study should not be taken as generalizable for other contexts since they focused on a specific IYCF program in a limited number of sub-districts in rural Bangladesh in which A&T had been implemented. This study would have been strengthened by conducting comparison focus groups in non-A&T areas of Bangladesh to explore CHW motivation in similar contexts; future research should also include comparison areas if resources allow. It is particularly important to note that the CHWs in the context of this study were unpaid, and performance-based financial incentives were the only type of financial compensation received. We are therefore unable to disentangle any unique effects that may be attributable to performance-based incentives, versus salaries, as a type of financial compensation. Similarly, cessation of trainings, performance monitoring procedures, and other A&T program features likely affected CHW motivation negatively, yet our data do not allow us to clearly separate the effects of withdrawing financial incentives from the effects of removing other types of support. Thus, our analysis focuses on instances where focus group participants explicitly mention the linkages between financial incentives and our conceptualization of motivation.

CHW supervisors who participated in the focus groups offered unique perspectives and insights, but they do not necessarily represent the actual opinions and perspectives of CHWs. Future research should seek to gather a broader range of perspectives from multiple types of stakeholders. Although the focus group facilitators made deliberate efforts to explain that the study was part of a learning exercise, it is possible that some supervisors’ saw the focus groups as an opportunity to advocate for more program funding, which may have biased their responses. Additional limitations should be considered due to the two-year timeframe that passed between the end of A&T and data collection for this study. It may be that the remaining CHWs were initially more motivated than those who had already left their positions at the time of the focus groups. Recall bias is another important consideration since multiple intervening factors (e.g., groupthink) could have played a role in participants’ views during the intervening period.

## Conclusion

Given the prominent role of CHWs in health systems around the world, improving CHW motivation and performance is a critical challenge to achieving the Sustainable Development Goals over the next decade [59]. Both to strengthen motivation and from a human rights and gender equity perspective, regular salaries are a critical element of CHW compensation structures. This study provides new evidence regarding how removing financial compensation from CHW programs can negatively affect CHW motivation. The findings lend support to the argument that, in order to sustain service coverage and quality, focused programs in Bangladesh and in other locations should carefully plan how CHWs will continue receiving compensation after conclusion of the program [38]. Although performance-based financial incentives can improve CHW motivation, these merit careful consideration by CHW project designers due to concerns about sustainability and negative effects. There is a need for more focused, high-quality research on the topic of incentives and motivation in order to explore whether performance-based incentives affect CHW motivation differently if their work is salaried or unpaid.

## Data Availability

The datasets analysed during the current study are available from the corresponding author on reasonable request.

## Abbreviations

A&T: Alive and Thrive
CHW: Community Health Worker
IYCF: Infant and Young Child Feeding
